# Interaction of differentiated human adipocytes with macrophages leads to trogocytosis and selective IL-6 secretion

**DOI:** 10.1038/cddis.2014.579

**Published:** 2015-01-22

**Authors:** A K Sárvári, Q-M Doan-Xuan, Z Bacsó, I Csomós, Z Balajthy, L Fésüs

**Affiliations:** 1 Department of Biochemistry and Molecular Biology, University of Debrecen, Debrecen, Hungary; 2 Department of Biophysics and Cell Biology, University of Debrecen, Debrecen, Hungary

## Abstract

Obesity leads to adipose tissue inflammation that is characterized by increased release of proinflammatory molecules and the recruitment of activated immune cells. Although macrophages are present in the highest number among the immune cells in obese adipose tissue, not much is known about their direct interaction with adipocytes. We have introduced an *ex vivo* experimental system to characterize the cellular interactions and the profile of secreted cytokines in cocultures of macrophages and human adipocytes differentiated from either mesenchymal stem cells or a preadipocyte cell line. As observed by time-lapse microscopy, flow, and laser-scanning cytometry, macrophages phagocytosed bites of adipocytes (trogocytosis), which led to their *de novo,* phagocytosis and NF-*κ*B-dependent synthesis, then release of interleukin (IL)-6 and monocyte chemoattractant protein (MCP)-1. IL-6 secretion was not accompanied by secretion of other proinflammatory cytokines, such as tumor necrosis factor (TNF)-*α* and IL-8, except MCP-1. LPS-induced release of TNF-*α*, IL-8 and MCP-1 was decreased in the presence of the differentiated adipocytes but the IL-6 level did not subside suggesting that phagocytosis-dependent IL-6 secretion may have significant regulatory function in the inflamed adipose tissue.

With weight gain in obesity a chronic low-grade inflammation develops associated with several metabolic diseases, such as type 2 diabetes mellitus, atherosclerosis and liver steatosis. This low-grade inflammatory response is mainly mediated by adipocytes regulating the release of adipocytokines, such as IL-6, TNF-*α* and MCP-1 that stimulates the infiltration of macrophages into adipose tissue and their activation.^1^ Adipocyte hypertrophy and local hypoxia are also implicated in macrophage recruitment, as the both conditions can mediate increased production of inflammatory cytokines and chemoattractants.^2^

White adipose tissue is characterized by a continuous turnover of the adipocytes with ~10% of annual renewal.^3^ Old cells usually die by apoptosis and are removed by professional phagocytes such as macrophages to keep cell number in a regulated equilibrium/balance.^4, 5^ It is generally accepted that apoptotic cells have a strong anti-inflammatory potential preventing inflammation in healthy tissue.^6^ The prevalence of macrophages in white adipose tissue of lean and obese mice and humans is selectively localized to dead adipocytes forming a so called crown-like structure.^7^ In lean adipose tissue the ratio of the macrophages is 5%, whereas, during obesity this rises up to 50%. The characteristics of macrophages are shifted toward a proinflammatory state in obese adipose tissue compared with lean individuals.^8, 9, 10^ Interestingly, the number of macrophages also increases during weight loss when adipocytes do not die, but shrink losing their lipid content.^11^

Although there is a direct contact between adipocytes and macrophages owing to the crown-like structure formation, not much is known about the consequences of these interactions. Most of the studies in the field rely on *in vivo* mouse model experiments, only a few cell culture observations were reported.^5, 12^ These studies reflected on the importance of cell–cell contact of adipocyte–macrophage interaction, which lead to proinflammatory cytokine secretion, such as IL-1*β*, TNF*α* and IL-6.^13, 14, 15^ A recently published review summarizes the knowledge on macrophage–adipocyte interaction highlighting the differences between the human and mouse adipose tissue biology and inflammation during obesity and points out the lack of sufficient information collected in human studies.^16^ Knowing that mouse and human macrophages differ with respect to their activation profiles,^17^ we developed a human *in vitro* experimental system to study cocultures of adipocytes and macrophages and learn what the outcome of their interaction is. We hypothesized that interaction between macrophages and adipocytes may lead to phagocytosis of the latter with significant consequences in the balance of pro- and anti-inflammatory factors. The presented results show that macrophages engulf pieces of living adipocytes through trogocytosis, which results in their selective IL-6 secretion with an anti-inflammatory effect.

## Results

### Pieces of differentiated adipocytes are phagocytosed by macrophages

**Figure 1:**
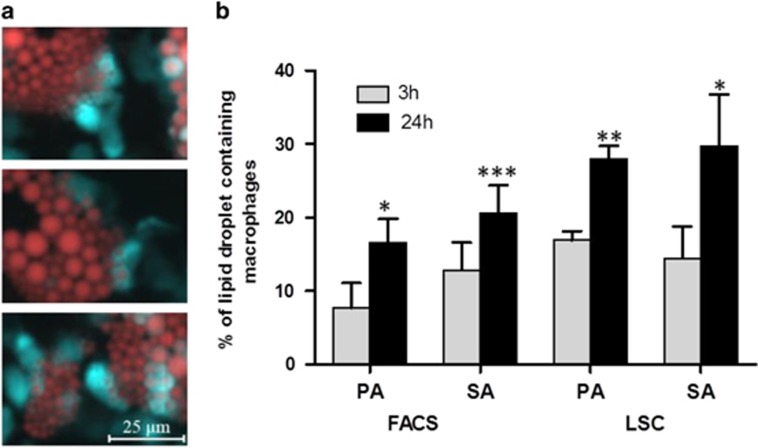
Detection of phagocytosis of differentiated adipocytes by macrophages (MФ). Adipocytes were prestained with 1 *μ*g/ml Nile red, MФs were prestained with 5 *μ*M CMFDA and cells were coincubated for 3 and 24 h. (**a**) Fluorescent microscopic images show MФs stained with CMFDA (cyan) which contain adipocyte-derived lipids stained with Nile red (red). (**b**) Red lipid droplets containing stained MФs were counted in whole-cell cocultures using laser-scanning cytometry. The phagocytosis ratio was determined by counting the percentage of lipid containing MФs by LSC. Data are expressed as mean±SD of three independent experiments, *P*-values are as follows; *P<0.05, **P<0.01 and ***P<0.001

To investigate whether macrophages could phagocytose adipocytes, we added macrophages to adipocytes differentiated in cell culture conditions. After their coincubation high proportion of macrophages contained lipid droplets (Figure 1a). The lipid containing macrophages were counted by flow or laser-scanning cytometery. Both analyses confirmed that macrophages efficiently engulf portions of adipocytes: after 3 h of coincubation ~15% of macrophages already contained lipid droplets, which increased up to 25–30% after 24 h (Figure 1b). Time-lapse microscopic images were taken to follow the process of phagocytosis; we could see several macrophages attacking one adipocyte (a much larger cell type) at a time and phagocytose pieces of the target cells (Supplementary Video 1).

### Interaction of macrophages and adipocytes leads to selective IL-6 secretion

**Figure 2:**
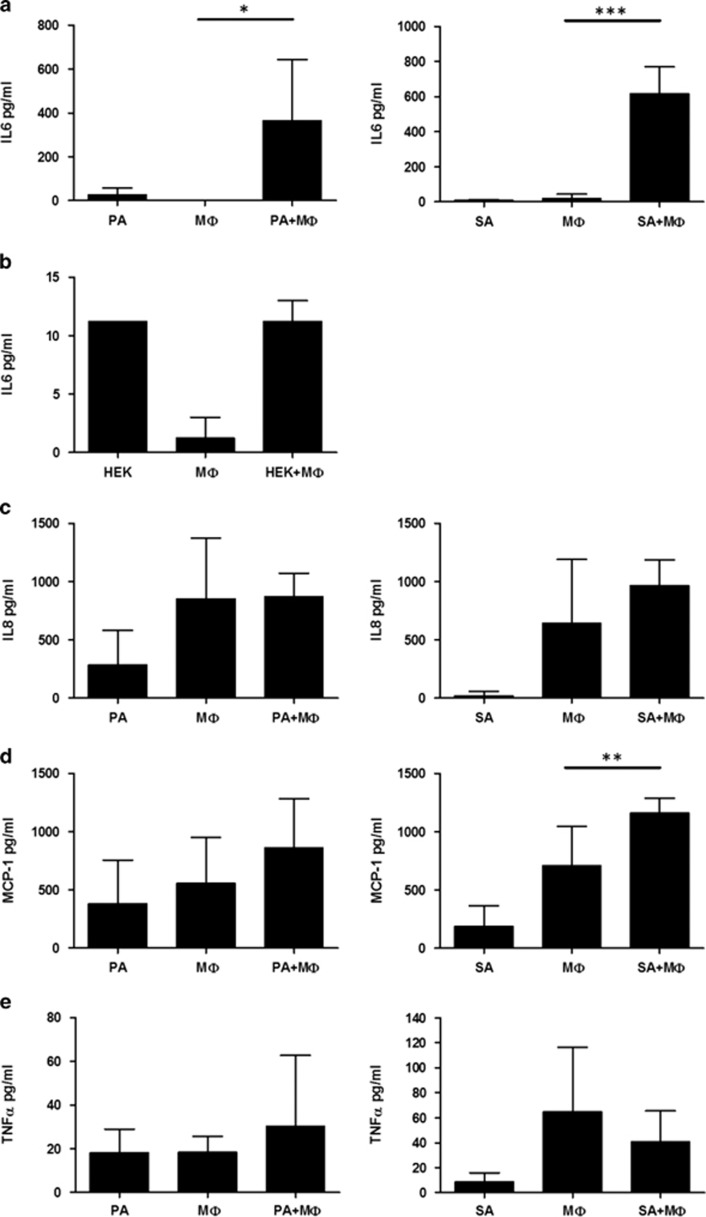
Interaction of macrophages and adipocytes leads to selective IL-6 secretion. Adipocytes (PA, SA) or HEK cells were coincubated with MФs for 12 h, then supernatants were collected and cytokine concentrations were measured by ELISA. Macrophages were added to the adipocytes or placed onto empty culture plates (MФ). Measurement of IL-6 secretion during co-incubation of MФ with PA or SA (**a**) and HEK cells (**b**). Secretion of IL-8 (**c**), MCP-1 (**d**) and TNF-*α* (**e**) during co-incubation of PA or SA and MФ. Data are expressed as mean±SD of five independent experiments. *P*-values are as follows; *P<0.05, **P<0.01 and ***P<0.001

Significant increase in IL-6 was detected in the culture media upon adipocyte–macrophage coincubation as compared with the basal level secreted by macrophages or adipocytes alone (Figure 2a). To prove that the adipocyte induced IL-6 production is a unique property of adipocyte–macrophage communication and not a response from the macrophage to not being allowed to attach to the surface of the plate, macrophages were plated onto another adherent cell type, namely HEK cells; this did not result in induction of IL-6 secretion (Figure 2b). The level of secreted IL-8 and IL-1*β* did not change (the latter was below the detection limit of the ELISA kit) (Figure 2c). MCP1 secretion was increased but it reached a significant level only when SGBS adipocytes were used (Figure 2d). Secretion of TNF*α* was not significantly induced during the coincubation (Figure 2e).

### IL-6 production in cocultures of adipocytes and macrophages depends on phagocytosis and is mediated by macrophages

**Figure 3:**
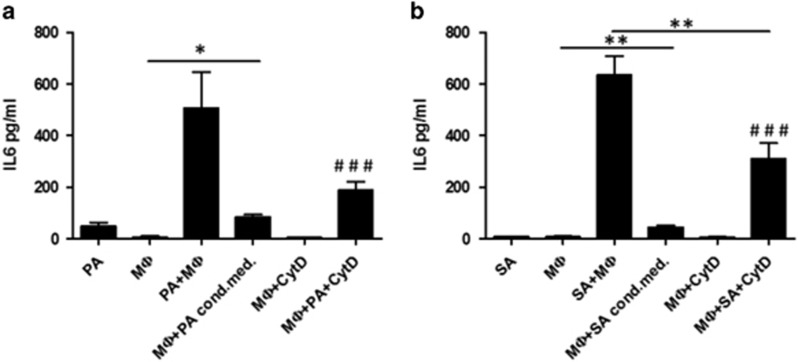
IL-6 secretion during adipocyte coincubation with MФs is phagocytosis dependent. MФs were cultured in adipocyte conditioned media or pretreated for 45 min with 20 *μ*M cytochalasin-D (CytD) before coculture with adipocytes. CytD concentration was maintained during coincubation as well. After 12 h supernatants of the cells were collected and the level of secreted IL-6 cytokine was measured by ELISA. Co-culture of MФ with PA (**a**) and SA (**b**). Data are expressed as mean±SD of five independent experiments. *P-*values are as follows; two-tailed paired *t*-test *P<0.05, **P<0.01; two-way ANOVA test ^###^*P*<0.001

To learn whether there is any secreted molecules originating from the adipocytes that induces the IL-6 secretion during coincubation, we cultured macrophages in adipocyte conditioned medium. This did not lead to an increased IL-6 secretion (Figures 3a and b) suggesting that the induction of IL-6 production is a consequence of interaction between adipocytes and macrophages.

To see whether the IL-6 secretion depends upon phagocytosis of adipocyte content, we blocked phagocytosis with CytD. CytD treatment attenuated IL-6 secretion, suggesting that is cell–cell contact was not enough to induce the same level of IL-6 secretion as in nontreated controls. (Figures 3a and b).

**Figure 4:**
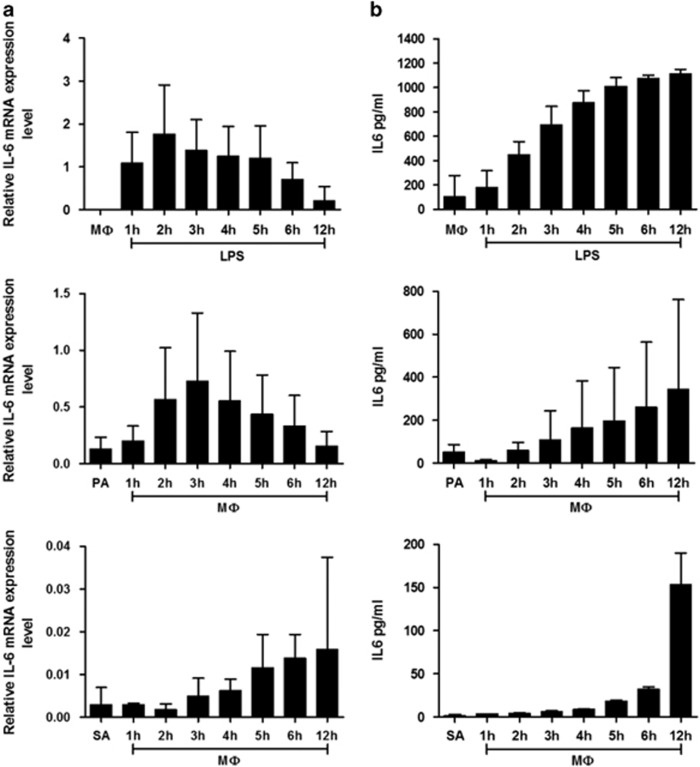
Relative gene expression and secreted protein levels in cultures of LPS-treated MФs and cocultures of adipocytes and macrophages. MФs were pretreated for 30 min with 0.5 *μ*g/ml crude LPS, then cells were cultured in fresh media for 12 h. Adipocytes were coincubated with MФs for 12 h. Relative mRNA expression of IL-6 (**a**) and levels of secreted IL-6 protein (**b**). Data are expressed as mean±SD of three independent experiments

Next, we compared the dynamics of the secretion and release of IL-6 after exposure of macrophages to either adipocytes or LPS. Time-dependent analysis of mRNA and secreted IL-6 shows the same pattern in the two cases (Figures 4a and b) suggesting that IL-6 is *de novo* synthesized during adipocyte–macrophage coincubation.

### IL-6 is produced by macrophages during adipocyte–macrophage interaction in an NF-*κ*B-dependent way

To further investigate which cells synthesized and secreted IL-6, brefeldin A (BrefA) was added to the cocultures of adipocytes and MΦs, and then cells were immunostained for IL-6. BrefA blocks protein transport through the Golgi, as well as, the endoplasmic reticulum and the produced IL-6 cytokine should accumulate in the cell.

**Figure 5:**
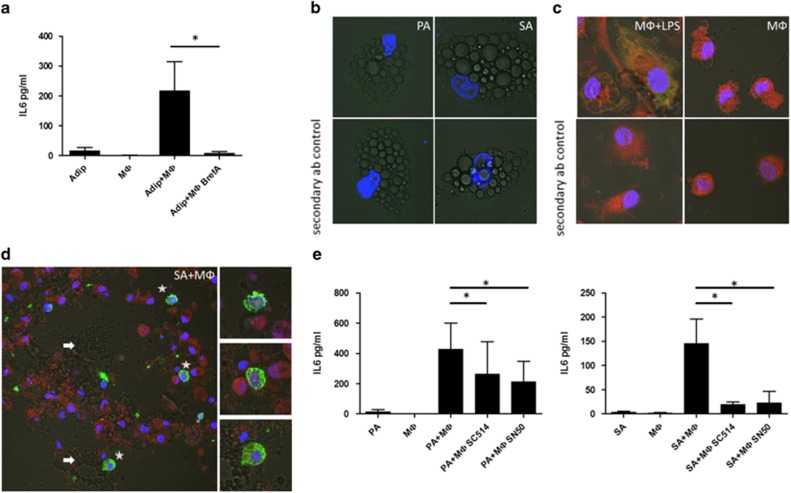
Immunostaining of IL-6 in MФ and the effect of Brefeldin A on IL-6 secretion. Cells were treated with 100 ng/ml brefeldin A (BrefA) to block the secretion of IL-6 during coincubation for 12 h. (**a**) BrefA treatment blocked IL-6 secretion during adipocyte and MФ co-incubation. Fluorescent confocal microscopic (**b**–**d**) images of MФs stained with CMTMR (red), of all cell types (MФ as well as adipocytes) stained with NucRed (blue) and immunostained for IL-6 (green). (**b**) Adipocytes alone; (**c**) LPS-treated and control macrophages were also treated with BrefA; (**d**) Adipocytes were coincubated with MФs and, after 12 h cells were fixed and immunostained. Arrows indicate adipocytes; asterisks indicate IL-6-producing MΦs, images on the side show six times magnified details of the original image. (**e**) Inhibitors of the NF-*κ*B pathway blunt IL-6 secretion during adipocyte–MФ coincubation. Hundred micromolar SC-514, or 50 mg/ml SN50 was applied to inhibit the NF-*κ*B pathway. After 12 h of coincubation the level of secreted IL-6 protein was measured as described previously. Data are expressed as mean±SD of three independent experiments. *P-*values are as follows; *P<0.05

Although there was no trace of IL-6 in BrefA-treated adipocytestes (Figures 5a and b), IL-6 accumulated in macrophages when the cocultures of adipocytes and macrophages were treated with BrefA (Figures 5a and d) similarly to LPS and BrefA-treated macrophages (Figure 5c). On the basis of these data it can be concluded that in adipocyte–macrophage interaction IL-6 is produced mostly by macrophages.

To detect, whether the adipocyte induced IL-6 secretion is mediated through NF-*κ*B signaling, NF-*κ*B pathway inhibitors were applied. SC-514 is a selective and reversible cell permeable inhibitor of IKK*β* (IKK-2), SN50 is a cell permeable peptide, which inhibits translocation of the NF-*κ*B active complex into the nucleus.

Both SC-514 and SN50 could decrease the IL-6 secretion during coincubation of PA or SA with macrophages (Figure 5e) indicating that IL-6 secretion is mediated through NF-*κ*B signaling.

### The influence of differentiated adipocytes on LPS-induced cytokine secretion of macrophages

**Figure 6:**
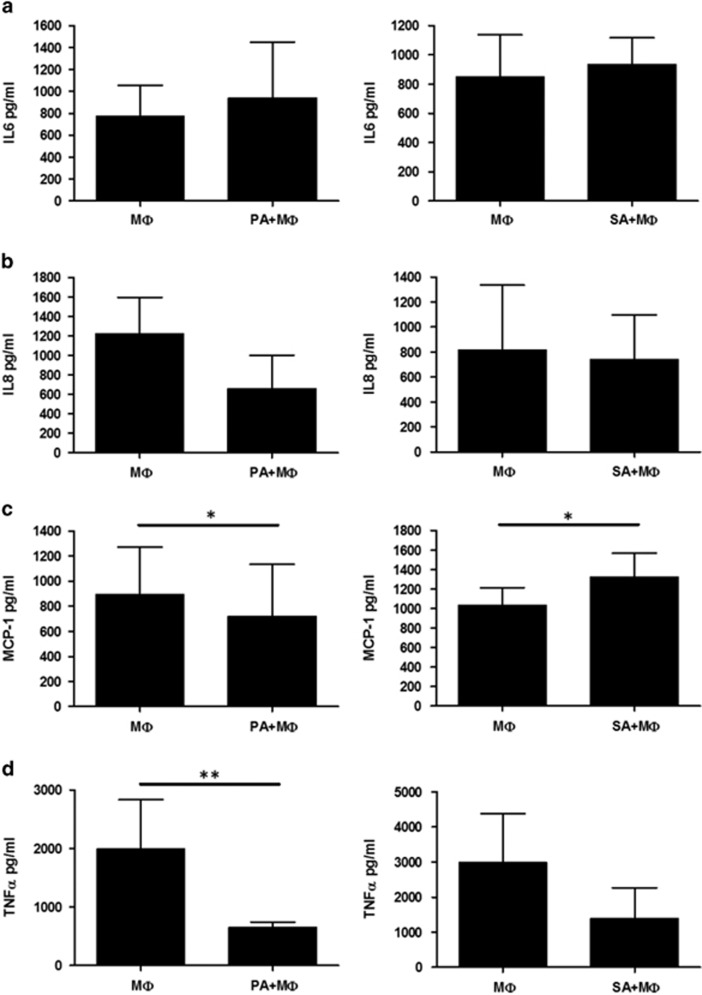
Influence of adipocytes on the secretion of inflammatory cytokines by LPS-treated macrophages. MФs were pretreated for 30 min with 0.5 *μ*g/ml crude LPS, then the activated macrophages were placed onto the culture plates or added to adipocytes (PA, SA) for 12 h. Supernatants were collected and the level of secreted cytokines was measured by ELISA method. Secretion of (**a**) IL-6, (**b**) IL-8, (**c**) MCP-1 and (**d**) TNF-*α* during co-incubation PA or SA and LPS treated MΦ. Data are expressed as mean±SD of five independent experiments. *P-*values are as follows; *P<0.05, **P<0.01

To check how ingested adipocyte material influences macrophages in an inflammatory environment, we pretreated macrophages with LPS before adding them to adipocytes. After coincubation, culture media were collected to measure the level of inflammatory cytokines (IL-6, IL-8, MCP1 and TNF*α*). Whereas the level of secreted IL-6 did not change (Figure 6a) in the presence of adipocytes, the amount of IL-8 has been decreased (Figure 6b) and the concentration of MCP1 and TNF*α* has been significantly reduced (Figures 6c and d).

## Discussion

While studying the interaction between human differentiated adipocytes and macrophages, we observed efficient phagocytosis of pieces of the adipocytes leading to the appearance of lipid drops in the macrophages. Adipocytes prepared from either adipose tissue-derived precursor cells or an established preadipocyte cell line, were consumed by the macrophages. The observed phagocytic process is not a typical apoptotic cell clearance phenomenon in which apoptotic cells are rapidly and completely engulfed by phagocytes to preserve tissue integrity and prevent release of potentially noxious or immunogenic intracellular materials from the dying cells.^18^ In our case the adipocytes did not seem to die, whereas macrophages took pieces out of them, and we did not see adipocyte-derived DNA in the phagocytes (data not shown). The phenomenon looks like trogocytosis, a process in which one cell takes bites out of another; this has previously been described among immune cells and proposed to serve as a way for cells to acquire nourishment from other cells.^19, 20^ We have previously reported that a significant portion of the differentiated human adipocytes have phosphatidylserine on their surface and contained partially fragmented DNA.^21^ A possible mediator of this special phagocytosis is the milk fat globule-EGF-factor 8 (MFG-E8) which is a secreted glycoprotein produced by activated macrophages, binds specifically to apoptotic cells by recognizing phosphatidylserine and attaches them to phagocytes for engulfment.^22^ The engulfment of apoptotic cells induces and activates PPAR-*δ*, which then further enhances the expression of opsonins, such as MFGE-8.^23^ The expression of MFGE-8 and the *α*_v_ and *β*_5_ integrin subunits are increased in adipose tissue of obese humans.^24^ A novel and possibly related role of MFGEf-8 has been recently revealed; it coordinates fatty-acid uptake through *α*_v_*β*_3_ integrin-and *α*_v_*β*_5_ integrin-dependent phosphorylation of Akt by phosphatidylinositide-3 kinase and mTOR complex2, leading to translocation of CD36 and Fatp1 from cytoplasmic vesicles to the cell surface. MFGE-8 promotes the absorption of dietary triglycerides and cellular uptake of fatty acids from blood stream.^25^ These findings raise the possibility of the potential involvement of MFGE-8 in the attraction of macrophages toward phosphatidylserine expressing differentiating adipocytes mediating the engulfment of adipocyte pieces.

Engulfment of apoptotic cells brings large amount of cellular lipids including oxidized fatty acids and oxysterols into the macrophage and PPARs are the sensors of the native and oxidized fatty acids, and the derivatives of the free fatty acids serve as hormonal ligand for PPAR*γ*.^26^ By sensing lipids from apoptotic cells, PPAR-*δ* functions as a molecular switch that discriminates between the proinflammatory and immunosuppressive actions of macrophages, it mediates the macrophage program of alternative activation.^23^ LXRs can respond to phagocytosed lipids and modulate apoptotic cell clearance and maintain immune tolerance trough transrepression of inflammation. In this context inhibition of inflammatory gene expression is linked to metabolism of apoptotic cell lipids.^26^ The engulfment and brake down of the lipid content of adipocytes by macrophages also may activate the PPAR and LXR transcriptional programs, further facilitating the phagocytosis and suppressing the proinflammatory reactions.

As an outcome of lipid engulfment during coincubation of adipocytes and macrophages, macrophages produced a high amount of IL-6, which was not accompanied by the induction of TNF-*α* and IL-1*β*. It has been previously demonstrated that IL-6 is released *in vivo* from human subcutaneous adipose tissue, whereas there was no TNF*-α* secretion from this depot;^27^ in this study the cellular source of IL-6 was not determined. Our immunostained images obtained in an experimental system made of human cells revealed that the source of IL-6 is the lipid droplet containing macrophage. This corroborates other studies, where it was shown that the macrophages are responsible for almost all IL-6 expression in adipose tissue.^8^ It cannot be excluded that different adipose tissue depots release different combination of cytokines.

The induction of IL-6 expression in our system was not mediated by the conditioned medium of the cultured adipocytes, which excluded the possibility that fatty acids or extracellular vesicles released from the adipocytes may mediate this phenomenon. It has been recently published that human adipocytes are capable to shed extracellular vesicles,^28^ which do not contain triacylglycerol but have been shown to have a role in signaling processes between adipocytes and monocytes. Some proteins, like adiponectin MIF and PRB4 have been shown to be partially released in exosomes from adipocytes and these proteins seem to have a role in macrophages differentiation, activation^29^ and subsequent development in insulin resistance.^30^ The extracellular vesicles, originated from *in vitro* differentiated SGBS adipocytes and human adipose tissue explants, upregulated the production of some proinflammatory (TNF-*α*, IL-6 and MIP-1*α*), as well as anti-inflammatory cytokine (IL-10). These experiments have been performed with monocytes and there is no data on the effect of these extracellular vesicles on mature macrophages. The discrepancy between these data and our results could be owing to the fact that in their study, Kranendonk *et al*^28^ collected the extracellular vesicles from adipocytes cultured for 48–72 h and then supplemented the monocytes with these samples through their differentiation from day 2. In our study we have collected adipocyte supernatant for 12 h and cultured the macrophages with it for another 12 h. It should be noted that in our coincubation experiments we have used 12 h coculture times, that is the formation and the effect of the extracellular vesicles might appear at a later state. Furthermore, the cytokine secretion shown in our study is due to removal and engulfment of triacylglycerol containing pieces of living adipocytes by macrophages.

FFA and other lipids have been found to regulate the activation state and immune function of macrophages; saturated fatty acids activate classical inflammatory responses in macrophages and other immune cells through engagement of pattern recognition receptors, including Toll-like receptors (TLRs).^15, 31^ However, IL-6 induction and secretion required phagocytosis of the lipid content of adipocytes and the digestion of triacylglycerol in lysosome could lead to release of fatty acids to lipid sensing TLRs, such as TLR4 (LPS serves as ligand), TLR1-2 heterodimer (liganded by triacyl lipopeptide) and TLR2-6 (activated by diacyl lipopeptide) which are located on the surface of the cells and in the cell membrane.^32, 33^ Saturated fatty acids are known to exert proinflammatory effects;^34^ lauric acid^35^ and palmitic acid^36^ released from dysregulated adipocytes can activate TLR-2 and TLR4 signaling, respectively, which ultimately triggers NF*κ*B mediated proinflammatory gene expression and subsequent cytokine secretion from macrophages. Macrophages activated through TLR2^35^ and TLR4^36^ signaling have been shown to undergo polarization to a unique M1-like phenotype characterized by increased lipid content and secretion of the proinflammatory cytokines TNF*-α* and IL-6.^15^ We could partially block the adipocyte-induced IL-6 secretion using different NF-*κ*B inhibitors, showing a possible involvement of TLR-dependent pathways in IL-6 secretion during coculture of adipocytes and macrophages.

Although both IL-6 and TNF-*α* are expressed by adipose tissue, it has been shown that there are important differences in their systemic release. TNF*-α* is not released by subcutaneous depot; in contrast, IL-6 is released from this depot and is thereby able to signal systemically. The release of IL-6 from subcutaneous depots into the systemic circulation and the fact that this release is greater on obese subjects support, a possible novel role for IL-6 as a systemic regulator of body weight and a regulator of lipid metabolism.^27^ Taking into consideration that leptin receptor shares homology with the gp130 signal-transducing component of the IL-6 receptor,^37^ IL-6 may modulate even the actions of adipocyte secreted leptin, which binds to hypothalamic receptors and regulates energy balance by causing changes in food intake, physical activity and thermogenesis.^27^

Whereas there is little doubt about the proinflammatory nature of TNF-*α* and IL-1*β* released during adipose tissue inflammation, IL-6 seems to be a pleiotropic cytokine, being able to act as pro- and anti-inflammatory regulator as well. During classical NF-*κ*B inflammatory pathway activation, TNF-*α* and IL-1*β* is secreted together with IL-6, but our results show an isolated IL-6 secretion when macrophages phagocytose pieces of adipocytes. It has been published that the endogenous IL-6 has a regulatory role in local acute inflammation and suppresses the proinflammatory cytokine synthesis, such as TNF-*α* and IL-1. IL-6 does not only negatively regulates the production of these cytokines, but it also induces the production of IL-1 and TNF antagonists in U937 cells.^38^ Furthermore, IL-6 can act to prime myeloid cells for IL-4 signaling during obesity in mice.^39^ As obese adipose tissue is described to be in an inflammatory state, we have checked the effect of adipocytes on cytokine production in an inflammatory environment using LPS-treated macrophages. The secretion of IL-6 was not influenced by adipocytes, but the levels of MCP-1 and TNF-*α* were significantly reduced during their coincubation with macrophages. Previously, it had been shown that IL-6 could inhibit LPS-induced TNF-*α* and IL-1*β* expression and secretion,^40^ and IL-6 limit LPS-induced endotoxemia in mice.^39^ On the basis of this data and our observation presented here, one may presume that IL-6 secreted during interaction of adipocytes and macrophages might have an anti-inflammatory role in the inflamed adipose tissue downregulating the induction and release of proinflammatory cytokines.

In summary, our results show a high level of *de novo* IL-6 secretion by macrophages as a result of engulfment of the lipid content of adipocytes by macrophages. Using a newly designed human *in vitro* experimental system, we could capture the interaction of macrophages and adipocytes in coculture. The absence of the secretion of the typical proinflammatory TNF-*α* and IL-1*β* and the selective appearance of the pleiotropic IL-6 shed a different light on the role of IL-6 in this interaction. Previous reports presented IL-6 as a key proinflammatory player in the inflamed obese adipose tissue. Our data suggest a possible positive role for IL-6 in suppressing the level of TNF-*α* and MCP-1, in maintaining adipose tissue homeostasis and in preventing the consequences of high proinflammatory cytokine levels, as insulin resistance and other elements of the metabolic syndrome.

## Materials and Methods

### Induction of adipocyte and macrophage differentiation

Adipocytes were differentiated from human adipose tissue-derived precursor cells, or from the Simpson–Golabi–Behmel syndrome (SGBS) preadipocyte cell line.^41^ Human adipose tissue was obtained from the subcutaneous adipose depot of volunteers undergoing herniectomy without other medical condition. Selection was made based on body mass index (<30), but not on age or gender. Informed consent was obtained from the subjects before the surgical procedure. The study protocol was approved by the Ethics Committee of the University of Debrecen, Hungary (No. 3186-2010/DEOEC RKEB/IKEB). The adipose tissue samples (1–10ml) were immediately transported to the laboratory after being removed. SGBS preadipocyte differentiation^41^ and adipose tissue-derived preadipocyte isolation and differentiation was performed according to the already described protocols.^42^

Human PBMCs were isolated by density gradient centrifugation on Ficoll-Paque Plus (Amersham Bioscience, Piscataway, NJ, USA) from ‘buffy coats' obtained from healthy blood donors. CD14^+^ cells were separtated by magnetic sorting with Vario-MACS (Miltenyi Biotech, Bergisch Gladbach, Germany), followed by washing with PBS containing 0.5% BSA and 2 mM EDTA. Freshly isolated monocytes were seeded into 24-well plates at a density of 10^6 ^cells/ml and cultured in IMDB medium (Sigma, St. Louis, MO, USA) suplemented with 10% of human AB serum (Sigma) and 5 nM MCSF (Bioscience/Promega, Madison, WI, USA) for 5 days to differentiate them to macrophages, the medium was refreshed after 3 days.

### Phagocytosis assay

Primary adipocytes (PA) differentiated for ten days and SGBS adipocytes (SA) were stained with Hoechst 33342 (Sigma, 50 *μ*g/ml) and 1 *μ*g/ml Nile red for 30 min. To decrease nonspecific accumulation of Hoechst and Nile red by macrophages during the phagocytosis process, cells were carefully washed two times in PBS. Macrophages were stained with fluorescent cell tracer green CMFDA (Invitrogen, Carlsbad, CA, USA) according to the manufacturer's protocol. Macrophages were layered on the top of adipocytes in a ratio of 5 : 1 and were cocultured for 24 h at 37 °C in a 5% CO_2_ atmosphere. Phagocytic ratio was determined counting the macrophages containing lipid droplets applying laser-scanning cytometry (LSC). For flow cytometric measurements, cells were trypsinized (Sigma) and centrifuged at 1800 rpm for 10 min; when macrophages sedimented to a pellet, whereas adipocytes, owing to their lipid content, remained in the supernatant. Cells in the pellet were examined by a FACSCalibur BD flow cytometer and list mode data were analyzed by WinMDI2.8 software (Freeware, written by Josef Trotter, downloaded from http://facs.scripps.edu).

### Laser-scanning cytometry (LSC)

Imaging cytometry measurements were performed by using iCys Research Imaging Cytometer (iCysTM, Thorlabs Imaging Systems, Sterling, VA, USA) equipped with 405-nm, 488-nm and 633-nm solid-state lasers, photodiode forward scatter detectors and photomultiplier tubes with three filters in front. Sample slides were mounted on the computer-controlled stepper motor-driven stage. An area of optimal confluency was selected in low-resolution scout scan with 10 × magnification objective (NA 0.30) and 10-*μ*m scanning step. High-resolution images of selected areas were obtained by using 40 × objective (NA 0.75) and 0.25-*μ*m step. Size of a pixel was set to 0.25 by 0.245 *μ*m at 40 × magnification. Laser lines were separately operated, namely 405-nm violet was used to excite Hoechst 33342 to recognize nuclei, and 488-nm blue line was used for CMFDA to recognize whole macrophages, and Nile red to identify lipid droplets. Emission of Hoechst was detected at 463±20 nm, CMFDA at 530±15 nm and Nile red at 580±15 nm. Images were processed and analyzed by an automatic cell recognition protocol developed by us utilizing iCys software (iNovator Application Development Toolkit, CompuCyte Corporation, Westwood, MA, USA), Image J (National Institute of Health, MD, USA)^43^ and CellProfiler (The Broad Institute of MIT, MA, USA),^44, 45^ as described earlier.^21^ Briefly, nuclei from both cell types were identified first and marked as primary objects. On the basis of parent nuclei, the secondary objects, e.g. whole macrophages and adipocytes, were subsequently recognized according to CFMFDA or Nile red signals. When macrophages have been segmented, image regions occupied by macrophages were excluded from further search for adipocytes. Phagocytotic ratio was determined counting macrophages containing Nile red stained lipid droplets.

### Time-lapse imaging microscopy

Adipocytes were stained with Nile red and Hoechst 33342, macrophages were layered on top of the adipocytes in a ratio of 5 : 1. The coculture was placed in a temperature-, humidity- and CO_2_-controlled, motorized Olympus IX-81 inverted microscope (Olympus America, Center Valley, PA, USA), which was equipped with a cooled Hamamatsu ORCA-R2 (Hamamatsu Photonics, Hamamatsu City, Japan) high-resolution monochrome CCD camera and a DP21-CU 2-megapixel digital color camera (Olympus). Cells were monitored for 15 h and in every 5 min an image was taken. Data were converted into a video file with the use of the Xcellence software (Olympus America).

### Determination of cytokine release

Differentiated adipocytes (PA and SA) were cocultured with macrophages for 12 h, and culture supernatants were collected and stored for cytokine measurements. In some experiments macrophages were treated with 100 *μ*M IKK-2 Inhibitor, SC-514 (Calbiochem, San Diego, CA, USA) or with 50 *μ*g/ml NF-*κ*B SN50 Cell-Permeable Inhibitor Peptide (Calbiochem) and cocultured with PA or SA. In further experiments, macrophages were pretreated with 50 ng/ml crud LPS for 30 min, or 20 *μ*M CytochalasinD (CytD) for 45 min and supernatant was collected after 12 h. The concentration of IL-6, IL-1*β*, IL-8, TNF*α* and MCP-1 was measured from the collected cell culture media using ELISA DuoSet (R&D Systems, Minneapolis, MN, USA).

### Immunostaining of intracellular IL-6 cytokine

Macrophages were prestained overnight with the cell tracer, CMTMR (Life Technologies). PA or SA and macrophages were cocultured with macrophages for 12 h in the presence of 100 ng/ml BrefeldinA (Sigma), a protein transport inhibitor. After coincubation, cells were fixed in 4% paraformaldehyde (Sigma), permeabilized with 0.1% triton X-100 (Sigma), blocked for 1 h with 5% horse serum (Gibco) containing milk powder and dissolved in 0.005% Tween-20 (Sigma) containing PBS. As a primary antibody, goat polyclonal anti-human IL-6 IgG (R&D Systems) was used in 200 × dilution for 2 h. As secondary antibody Anti-Goat IgG–FITC, produced in rabbit (Sigma) was applied in a 500 × dilution for 1 h. Olympus FluoView 1000 Confocal microscope (Melville, NY, USA) was used to detect the localization of IL-6 (FITC 488 nm), macrophages (CMTMR 546 nm) and the nucleus (NucRed 647 nm). For the excitation of FITC-labeled secondary antibody the 488 nm line of an Argon ion laser; for CMTMR a 543-nm He–Ne laser; for NucRed a 633-nm He–Ne laser was used. Fluorescence emissions were detected through 500–530-nm, 555–625-nm and 655–755-nm band-pass filters, respectively.

### Real-time Q-PCR

PA, SA were coincubated with macrophages, macrophages were pretreated for 30 min with 50 ng/ml crude LPS. Cells were collected after 1, 2, 3, 4, 5, 6 and 12 h in 1 ml Tri Reagent (Invitrogen) for total RNA isolation and reverse transcribed to cDNA by High-Capacity Reverse Transcription kit (Applied Biosystems, Carlsbad, CA, USA) according to the manufacturers' instructions. Transcript levels of IL-6 were determined by real time Q-PCR using TaqMan Gene Expression Assay (Applied Biosystems). Samples were measured in three technical parallels. Genes were normalized for GAPDH housekeeping gene.

### Statistical analysis

For the statistical analyses, two-tailed paired *t*-test (*) and two-way ANOVA test (#) was applied.

## Abbreviations

BrefA: Brefeldin A
CytD: CytochalasinD
IL: Interleukin
LSC: Laser-scanning cytometry
MCP: Monocyte chemoattractant protein
MFG-E8: Milk fat globule-EGF-factor 8
MФ: Macrophages
PA: Primary adipocytes
SA: SGBS adipocytes
SGBS: Simpson–Golabi–Behmel syndrome
TLRs: Toll-like receptors
TNF: Tumor necrosis factor
